# Ochratoxin A Status at Birth Is Associated with Reduced Birth Weight and Ponderal Index in Rural Burkina Faso

**DOI:** 10.1016/j.tjnut.2024.10.015

**Published:** 2024-10-10

**Authors:** Yuri Bastos-Moreira, Alemayehu Argaw, Giulianmichela Di Palma, Trenton Dailey-Chwalibóg, Jasmin El-Hafi, Lionel Olivier Ouédraogo, Laeticia Celine Toe, Sarah De Saeger, Carl Lachat, Marthe De Boevre

**Affiliations:** 1 Center of Excellence in Mycotoxicology and Public Health, MYTOX-SOUTH® Coordination Unit, Faculty of Pharmaceutical Sciences, Ghent University, Ghent, Belgium; 2 Department of Food Technology, Safety and Health, Faculty of Bioscience Engineering, Ghent University, Ghent, Belgium; 3 Department of Chemistry, University of Torino, Torino, Italy; 4 Institute of Food Chemistry, University of Münster, Münster, Germany; 5 Laboratoire de Biologie Clinique, Centre Muraz, Bobo-Dioulasso, Burkina Faso; 6 Unité Nutrition et Maladies Métaboliques, Institut de Recherche en Sciences de la Santé (IRSS), Bobo-Dioulasso, Burkina Faso; 7 Department of Biotechnology and Food Technology, Faculty of Science, University of Johannesburg, Doornfontein Campus, Gauteng, South Africa

**Keywords:** birth outcomes, exposomics, growth, low- and middle-income countries, MISAME-III, mycotoxins, sub-Saharan Africa, ochratoxin A

## Abstract

**Background:**

Mycotoxin exposure during pregnancy has been associated with adverse birth outcomes and poor infant growth. We assessed multiple biomarkers and metabolites of exposure to mycotoxins at birth and their associations with birth outcomes and infant growth in 274 newborns in rural Burkina Faso.

**Methods:**

Whole-blood microsamples were analyzed for mycotoxin concentrations in newborns in the Biospecimen substudy nested in the MIcronutriments pour la SAnté de la Mère et de l’Enfant-III trial using ultra-performance liquid chromatography coupled with tandem mass spectrometry. Unadjusted and adjusted associations between mycotoxin exposure, and birth outcomes and infant growth at 6 mo were estimated using linear regression models for continuous outcomes and linear probability models with robust variance estimation for binary outcomes. Infant growth trajectories from birth to 6 mo were compared by exposure status using mixed-effects models with a random intercept for the individual infant and a random slope for the infant’s age.

**Results:**

Ochratoxin A (OTA) exposure was detected in 38.3% of newborns, with other mycotoxins being detected in the range of 0.36% and 4.01%. OTA exposure was significantly associated with adverse birth outcomes, such as lower birth weight [β (95% CI): −0.11 kg (−0.21, 0.00); *P* = 0.042] and ponderal index [β (95% CI): −0.62 gm/cm^3^ (−1.19, −0.05); *P* = 0.034], and a marginally significant lower length growth trajectories during the first 6 mo [β (95% CI): −0.08 cm/mo (−0.15, 0.0); *P* = 0.057].

**Conclusions:**

OTA exposure was prevalent among newborns and also associated with lower growth at birth and during the first 6 mo. The results emphasize the importance of nutrition-sensitive strategies to mitigate dietary OTA, as well as adopting food safety measures in Burkina Faso during the fetal period of development.

Mycotoxins are toxic fungal secondary metabolites that contaminate many essential foods worldwide, including staple crops consumed by the most vulnerable populations [1]. Foodstuffs in West Africa are commonly affected by mycotoxins [2] because the climate is characterized by high temperature and humidity, creating favorable conditions for their production [3]. Maternal nutrition affects the pregnancy’s process and the newborn’s well-being [4]. In low- and middle-income countries (LMICs), adverse pregnancy outcomes are common including low birth weight (LBW), preterm birth (PTB), and/or small-for-gestational age (SGA) [5]. Several epidemiologic studies have indicated that mycotoxin exposure is extensive in newborns [[6], [7], [8]].

The International Agency for Research on Cancer classifies aflatoxin B1 (AFB1), aflatoxin B2 (AFB2), aflatoxin G1 (AFG1), and aflatoxin G2 (AFG2) as carcinogenic to humans (group 1). Additionally, aflatoxin M1 (AFM1), fumonisin B1 (FB1), fumonisin B2 (FB2), and ochratoxin A (OTA) are categorized as possible human carcinogens (group 2B) [9]. The human fetus is vulnerable to health effects resulting from *in utero* exposure to environmental chemicals [10]. Formerly, higher aflatoxin (AF) exposure, *in utero* and in early life, has been linked with stunting and/or underweight, whereas children with high fumonisins exposure were also shorter and lighter [11] Furthermore, research has shown that once OTA is absorbed from the gastrointestinal tract, it primarily binds to albumin with high affinity, resulting in a long half-life of 35.6 d [12,13]. OTA can cross the placental barrier in humans and has been linked to reduced birth weight in newborns [14,15], whereas animal studies have reported hemorrhages, maternal deaths, and reductions in both maternal and fetal body weights [16]. However, further studies are needed, because some research did not find an association between OTA exposure and birth anthropometry [17].

A literature review by Arce-López et al. (2020) [13] concluded that OTA is often detected in whole-blood, plasma, and serum samples. A meta-analysis of the literature results reported frequency levels of 64.9% [[18], [19], [20], [21]] and concluded that the global population is generally exposed to OTA due to its long half-life in these matrices [13]. This exposure during the critical first 1000 d of life [7] might contribute to adverse fetal and infant outcomes [22]. Generally, birth weight is an indicator of both maternal health and nutrition status, and also the infant’s well-being. Infants born with LBW are at increased risk of several short- and long-term consequences, including neonatal mortality, childhood stunting, and impaired immune function [[23], [24], [25]]. Nevertheless, studies investigating the association between mycotoxins exposure and birth and infant growth outcomes have reported inconsistent results [[26], [27], [28], [29]].

In Burkina Faso, limited biological and toxicologic food contamination data are available [30], and legislation and regulations regarding mycotoxins are often not implemented [31]. Using data from the Biospecimen (BioSpé) substudy of the MISAME-III (MIcronutriments pour la SAnté de la Mère et de l’Enfant) trial in rural Burkina Faso, we previously reported a prenatal exposure to multiple mycotoxins among pregnant women from a rural Burkinabé setting and found no evidence of associations with adverse birth outcomes and infant growth [32]. In the present study, we aimed to quantify newborn mycotoxin exposure at birth and investigated the association with birth outcomes and infant growth in the same mother–newborn dyads.

## Methods

### Study setting, participants, and design

**FIGURE 1:**
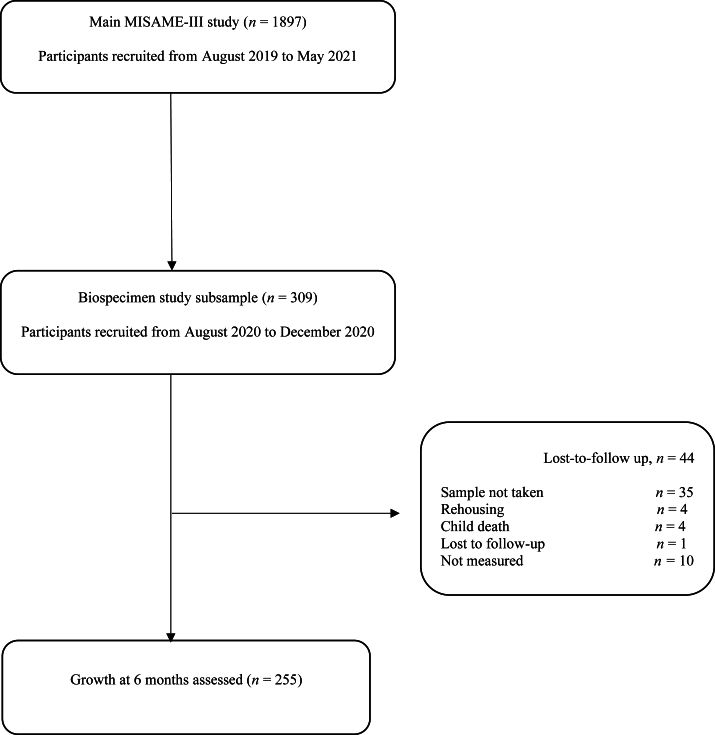
Study flow diagram of the Biospecimen sub-study (BioSpé) of the MISAME-III project. MISAME-III; MIcronutriments pour la SAnté de la Mère et de l’Enfant III.

Study protocols for the main MISAME-III trial [33] and the BioSpé substudy nested under the MISAME-III trial [34] were published previously. The main MISAME-III study is a 2 × 2 factorial randomized controlled trial evaluating the effect of balanced energy-protein (BEP) supplementation to mothers during pregnancy (prenatal intervention) and lactation (postnatal intervention) on maternal and child outcomes. Pregnant and lactating women aged between 15 and 40 y who lived in the study villages were identified through a census conducted in the research area (*n* = 10,165). Community support staff visited all eligible participants at their residences every 5 wk to identify early pregnancy by screening for self-reported amenorrhea. Women who were suspected of being pregnant were guided to the health center for a urine pregnancy test, and pregnancies were confirmed via ultrasounds. The participants excluded from the study included those who planned to leave the study area during pregnancy or deliver outside the study area and individuals who had a peanut allergy because the BEP is an energy-dense peanut paste [33]. In a subsample of 309 pregnant women, who were the last to enroll in the main MISAME-III trial (Figure 1), a BioSpé substudy was conducted. This substudy aimed to understand the physiologic mechanisms through which the BEP supplement affects maternal health, birth outcomes, and infant growth by way of multiomics analyses, human biomonitoring of contaminants (mycotoxins, black carbon, gutenteropathogens, and pesticides), and analysis of relative telomere length and mitochondrial DNA content [34].

The study was conducted in 6 rural health center catchment areas in the district of Houndé in the Hauts-Bassins region of Burkina Faso. The study area is characterized by a Sudano-Sahelian climate with a dry season running between September/October and April, and agricultural activities being the main livelihood of the community. Results from a previously conducted dietary survey in a subsample of MISAME-III pregnant women showed the habitual diet during pregnancy is nondiverse, predominantly based on maize with a complement of leafy vegetables [35]. Grains, roots, tubers, and plantains together contributed 68% of the total energy intake during pregnancy. Almost all participants (95%) consumed the main staple dish tô, which contributed 42% of the total energy intake. Tô is a stiff maize dough often served with a watery sauce containing green-leafy vegetables (okra, hibiscus, and baobab leaves) or other vegetables such as eggplant, with or without meat, fish, or caterpillars. Other food groups such as fruits, dairy, eggs, fish, and meat contributed very small amounts to the total energy intake [36].

### Exposure and outcomes

The present study considered exposure to 35 mycotoxins listed in Table 1, including AFB1, deoxynivalenol (DON), FB1, OTA, and zearalenone (ZEN). However, AFB1-lysine exposure was not assessed due to the current lack of a commercial analytical standard for this specific mycotoxin. Mycotoxin exposure was defined as the detection of a concentration ≥ the limit of detection (LOD) in whole-blood microsamples.

**TABLE 1:** Newborn mycotoxin exposure at birth.

Mycotoxins	Positive samples: *n* (%)	LOD	LLOQ
15-Acetyldeoxynivalenol	0.00	2.1	3.9
3-Acetyldeoxynivalenol	0.00	0.9	1.7
Aflatoxin B1	1 (0.36)	0.06	0.12
Aflatoxin B2	0.00	0.09	0.16
Aflatoxin G1	6 (2.19)	0.05	0.09
Aflatoxin G2	0.00	0.08	0.15
Aflatoxin M1	2 (0.73)	0.11	0.20
Alpha-zearalenol	0.00	4.0	7.4
Alternariol	0.00	1.2	2.3
Alternariol monomethyl ether	0.00	1.0	1.9
Beauvericin	0.00	0.8	1.4
Beta-zearalenol	0.00	2.9	5.3
Citrinin	3 (1.1)	2.6	4.7
Cyclopiazonic acid	0.00	1.44	2.65
Deepoxy-deoxynivalenol	0.00	0.70	1.40
Deoxynivalenol-3-glucoside	0.00	2.1	3.8
Deoxynivalenol	2 (0.73)	0.40	0.80
Diacetoxyscirpenol	0.00	0.9	1.6
Enniatin A	0.00	1.05	1.93
Enniatin A1	0.00	1.00	1.84
Enniatin B	0.00	0.92	1.69
Enniatin B1	0.00	0.92	1.69
Fumonisin B1	0.00	1.8	3.4
Fumonisin B2	0.00	2.0	3.7
Fumonisin B3	0.00	2.0	3.6
Fusarenone-X	0.00	0.7	1.3
Neosolaniol	0.00	0.80	1.50
Nivalenol	0.00	1.5	2.7
Ochratoxin A	105 (38.32)	0.09	0.17
Ochratoxin alpha	0.00	0.05	0.09
Roquefortine-C	0.00	0.9	1.6
Sterigmatocystine	0.00	0.8	1.5
T-2-toxin	0.00	0.59	1.1
Zearalanone	1 (0.36)	0.4	0.74
Zearalenone	11 (4.01)	0.70	1.30

Abbreviations: LOD, limit of detection; LLOQ, lower limit of quantification.

The outcomes of interest were birth outcomes, such as birth weight, SGA, LBW, gestational age (GA), PTB, length, mid-upper arm circumference (MUAC), head circumference, ponderal index, chest circumference, and infant growth and nutritional status at the age of 6 mo, such as length-for-age *z*-score (LAZ), weight-for-age *z*-score (WAZ), weight-for-length *z*-score (WLZ), MUAC, head circumference, hemoglobin, stunting, underweight, wasting and anemia. We additionally assessed the associations between mycotoxin exposure and infant growth trajectories (length, weight, upper arm, and head circumferences) during the first 6-mo postpartum.

PTB was defined as the birth of a newborn before 37 completed weeks of gestation. SGA was defined as a newborn weight less than the 10th percentile of weight for the same GA and sex according to the International Fetal and Newborn Growth Consortium for the 21st Century [37]. Anthropometric *z*-scores of LAZ, WAZ, and WLZ were calculated based on the WHO Child Growth Standards with stunting, underweight, and wasting defined as LAZ, WAZ, and WLZ values < 2 SD from the median value for same age and sex from the reference population [38]. Newborn Rohrer’s ponderal index was calculated as weight in g divided by length in cm cubed (i.e., weight/length^3^ (g/cm^3^) × 1000).

### Data collection

The MISAME-III trial data were collected through computer-assisted personal interviewing using SurveySolutions (version 21.5) on tablets and then transferred to a central server at Ghent University. Sociodemographic and other relevant characteristics of participants and study households were collected at baseline during the first and early second trimester of pregnancy. All newborn anthropometry measurements were taken within 12 h of birth, whereas mothers were invited for follow-up growth assessment every month until 6 mo of age. Measurements were taken in duplicates and a third measurement was taken in case of a large discrepancy between the duplicate measurements. Length was measured to the nearest 1 mm with a Seca 416 Infantometer, weight was measured to the nearest 10 g with a Seca 384 scale, and head circumference, thoracic circumference, and MUAC were measured to the nearest 1 mm with a Seca 212 measuring tape. GA was determined using a portable ultrasound (SonoSite M-Turbo, FUJIFILM SonoSite, Bothell) during the first and early second trimester of pregnancy.

### Blood sample collection and laboratory analysis

Samples collection and laboratory analysis procedures were described in detail previously [34]. Newborn samples were collected between May and October 2021 within 12 h of birth in all newborns. A volume of 40 μL of capillary whole blood was collected by capillary sampling onto volumetric absorptive microsampling (VAMS) tips (2 × 20 μL VAMS tips), namely Mitra, via heel-prick using BD Quikheel preemie lancet (0.85 mm × 1.75 mm, BD, Franklin Lakes) [34]. Following WHO guidelines, the sides of the heel are selected as the sampling site for neonatal participants, with the prick depth carefully limited to 2.4 mm or less to prevent nerve damage and skin breakdown due to adhesive strips. [39].

Then, VAMS tips were stored in 20 μL Mitra Clamshells and transported from the health centers to the Institut de Recherche en Sciences de la Santé in Bobo-Dioulasso, Burkina Faso for shipment at room temperature to the Centre of Excellence in Mycotoxicology and Public Health, Faculty of Pharmaceutical Sciences, Ghent University, Belgium. For storage at −80°C until analysis, VAMS were placed in Mitra Autoracks (96-Sampler, item number: 108) inside a storage bag containing desiccant bags (item number: AC-SS02). Items used for the VAMS collection were purchased from Neoteryx.

A VAMS multi-mycotoxin extraction [40] began by transferring the VAMS tips from the plastic handles into 2 mL Eppendorf tubes, and pipetting 250 μL extraction solvent (acetonitrile/water/acetic acid, 59/40/1, *v*/*v*/*v*), containing the internal standards ^13^C_17_–AFB1 (0.125 μg/L) and ^13^C_15_ – DON (0.25 μg/L), ^13^C_34_–FB1 (0.25 μg/L) and ^13^C_18_– ZEN (0.125 μg/L), to the sample tubes. Subsequently, samples were ultrasonicated for 30 min and shaken for 60 min at 25°C with rotation at 1400 × *g* in a Biosan TS-100 Thermo-Shaker followed by centrifugation (10 min at 10,000 × *g*, room temperature). The tips were discarded, and the supernatant was pipetted to an 8-mL glass tube and evaporated under nitrogen on a Turbovap LV Evaporator. Next, the extracts were reconstituted in 50 μL of injection solvent (methanol/water, 60/40, *v*/*v*), vortexed, centrifuged (for 10 min at 5000 × g, room temperature), and filtered (22 μm, PVDF, Durapore). Lastly, samples were transferred into vials before 10 μL were injected into an Acquity ultrahigh performance liquid chromatography (UPLC) system (Waters equipped with an Acquity HSS T3 100 × 2.1 mm UPLC column (1.8 μm particle size) and Acquity Vanguard HSS T3 10 × 2.1 mm UPLC pre-column (1.8 μm particle size), both from Waters. Detailed instrument parameters can be found in a previous study [40].

### Statistical analysis

Data management and statistical analyses were performed using Stata (Stata Statistical Software: Release 17.0; StataCorp), and a 2-sided statistical significance was considered at *P <* 0.05. Descriptive statistics are presented using means ± SD or medians (range) for the continuous variables, depending on the nature of the data distribution, and frequencies (percentages) for nominal variables.

In the study sample, only exposure to OTA was found in an adequate number of newborns to assess the association with birth outcomes and infant growth. The association between OTA exposure and the study outcomes at birth and 6 mo of age was evaluated using linear regression models for the continuous outcomes and linear probability models with robust variance estimation for the binary outcomes. All models were adjusted for clustering by the health center catchment areas and allocation for the prenatal and postnatal BEP interventions. Furthermore, adjusted models additionally included the covariates maternal age, primiparity, baseline BMI and hemoglobin concentration, household size, wealth index score, access to improved water and sanitation, and food security status.

We also compared OTA-exposed and nonexposed groups by growth trajectories from birth to 6 mo. For this purpose, we fitted mixed-effects regression models with random intercept for the individual infant and a random slope for the infant’s age (months). We explored the best growth trajectory fitting the data by visual inspection of graphs and comparing model fit indices including Akaike Information Criterion and Bayesian Information Criterion values. Accordingly, we applied quadratic models (for the outcome length, weight, and MUAC) and a restricted cubic spline model with 4 knots (for the outcome head circumference). We considered an unstructured covariance matrix for the correlation among repeated measurements within an individual. Fixed effects in the model included the main effect of OTA exposure, the main effect of age, and exposure by age interaction, which the latter estimates the difference in monthly changes in the outcome between exposure and unexposed groups. Models were further adjusted for the aforementioned covariates.

In a further exploratory analysis, we evaluated potential interactions between OTA exposure and the allocation of maternal BEP interventions on the study outcomes. For this purpose, interaction terms between OTA exposure and the prenatal and postnatal BEP interventions were specified in the models with the presence of interaction considered at *P <* 0.10. Lastly, Cohen’s weighted kappa test was used to assess the level of agreement between mother–newborn OTA exposure status. Results were reported as percentage agreement and Cohen’s weighted kappa values. The following cutoffs were used: Kappa values ≤ 0, no agreement; 0.01–0.20, none to slight; 0.21–0.40, fair; 0.41– 0.60, moderate; 0.61–0.80, substantial; and 0.81–1.00, almost perfect agreement [41].

## Results

### Mother–newborn dyads characteristics

Birth outcomes and infant growth at 6 mo were assessed on 274 and 255 newborns, respectively (Figure 1). The mean ± SD age of the mothers was 24.3 ± 5.63 y and 45.3% of mothers had at least a primary education (Table 2). The mean ± SD maternal BMI at study inclusion was 22.1 ± 3.22 kg/m^2^ with 6.93% underweight (BMI < 18.5 kg/m^2^). More than two-thirds (70.3%) of the newborns were from food-insecure households and 29.9% of their mothers were anemic at study enrollment during the first/early second trimester of pregnancy.

**TABLE 2:** Characteristics of study participants.

Characteristics	All subjects (*n* = 274)	OTA unexposed (*n* = 169)	OTA exposed (*n* = 105)
Study health center catchment area			
Boni	43 (15.7)	29 (17.2)	14 (13.3)
Dohoun	35 (12.8)	20 (11.8)	15 (14.3)
Dougoumato II	46 (16.8)	22 (13.0)	24 (22.9)
Karaba	37 (13.5)	18 (10.7)	19 (18.1)
Kari	58 (21.2)	41 (24.3)	17 (16.2)
Koumbia	55 (20.1)	39 (23.1)	16 (15.2)
Ethnic group			
Bwaba	156 (56.9)	99 (58.6)	57 (54.3)
Mossi	88 (32.1)	54 (32.0)	34 (32.4)
Others	30 (11.0)	16 (9.4)	14 (13.3)
Religion pregnant women			
Muslim	119 (43.4)	71 (42.0)	48 (45.7)
Protestant	64 (23.4)	35 (20.7)	29 (27.6)
Animist	62 (22.6)	48 (28.4)	14 (13.3)
Catholic	22 (8.0)	11 (6.5)	11 (10.5)
No religion, no animist	5 (1.8)	3 (1.8)	2 (1.9)
Primary education and above	124 (45.3)	80 (48.2)	44 (41.9)
Household food insecurity^1^	192 (70.3)	111(65.7)	81 (77.9)
Improved primary water source^2^	162 (59.1)	102 (60.4)	60 (57.1)
Improved sanitation facility^3^	174 (63.5)	105 (62.1)	69 (65.7)
Trimester of pregnancy at enrollment			
First	219 (79.9)	139 (82.2)	80 (76.2)
Second	55 (20.1)	30 (17.8)	25 (23.8)
Parity			
0	69 (25.2)	38 (22.5)	31 (29.5)
1–2	105 (38.3)	76 (45.0)	29 (27.6)
≥3	100 (36.5)	55 (32.5)	45 (42.9)
Wealth index, 0–10 points	4.70 ± 1.78	4.80 ± 1.75	4.54 ± 1.83
Household size	6.47 ± 4.64	6.64 ± 4.75	6.18 ± 4.47
Age, (y)	24.26 ± 5.63		
Weight, (kg)	58.40 ± 9.67	59.24 ± 9.81	57.03 ± 9.32
Length, (cm)	162.47 ± 5.73	163.01 ± 5.35	161.61 ± 6.23
BMI, (kg/m^2^)	22.09 ± 3.22	22.27 ± 3.34	21.79 ± 3.01
MUAC, (mm)	261.6 ± 27.9	262.6 ± 28.8	260.0 ± 26.3
Hemoglobin, (g/dL)	11.69 ± 1.45	11.76 ± 1.42	11.58 ± 1.49
Maternal prenatal supplementation			
BEP + IFA	142 (46.0)	81 (47.9)	44 (41.9)
IFA	167 (54.1)	88 (52.1)	61 (58.1)
Maternal postnatal supplementation			
BEP + IFA	156 (50.5)	94 (55.6)	50 (47.6)
IFA	153 (49.5)	75 (44.4)	55 (52.4)

Data are frequencies (%) or means ± SD.

Abbreviations: BEP, balanced energy-protein; IFA, iron-folic acid; HB, hemoglobin; MUAC, mid-upper arm circumference; OTA, ochratoxin A.

1 Assessed using Food and Nutrition Technical Assistance III Project/United States Agency for International Development’s Household Food Insecurity Access Scale.

2 Protected well, borehole, pipe, or bottled water were considered improved water sources.

3 Flush toilet connected to local sewage or septic tank or pit latrine with slab and/or ventilation was considered improved sanitation facilities.

### Mycotoxins exposure and newborn and infant growth and nutritional status

The laboratory analysis indicated that, aside from OTA, almost all newborns were found to be not exposed to most mycotoxins (Table 1). OTA exposure was detected in 38.3% of the newborns with a median (range) concentration of <LOD (<LOD, 3.61) μg/L. The LOD for OTA is 0.09 μg/L.

Other mycotoxins such as AFB1, AFG1, AFM1, DON, citrinin (CIT), zearalanone, and ZEN were detected in the range of 0.36% and 4.01% of newborns. For the remaining 26 mycotoxins analyzed, no exposure was detected through whole-blood analysis.

**FIGURE 2:**
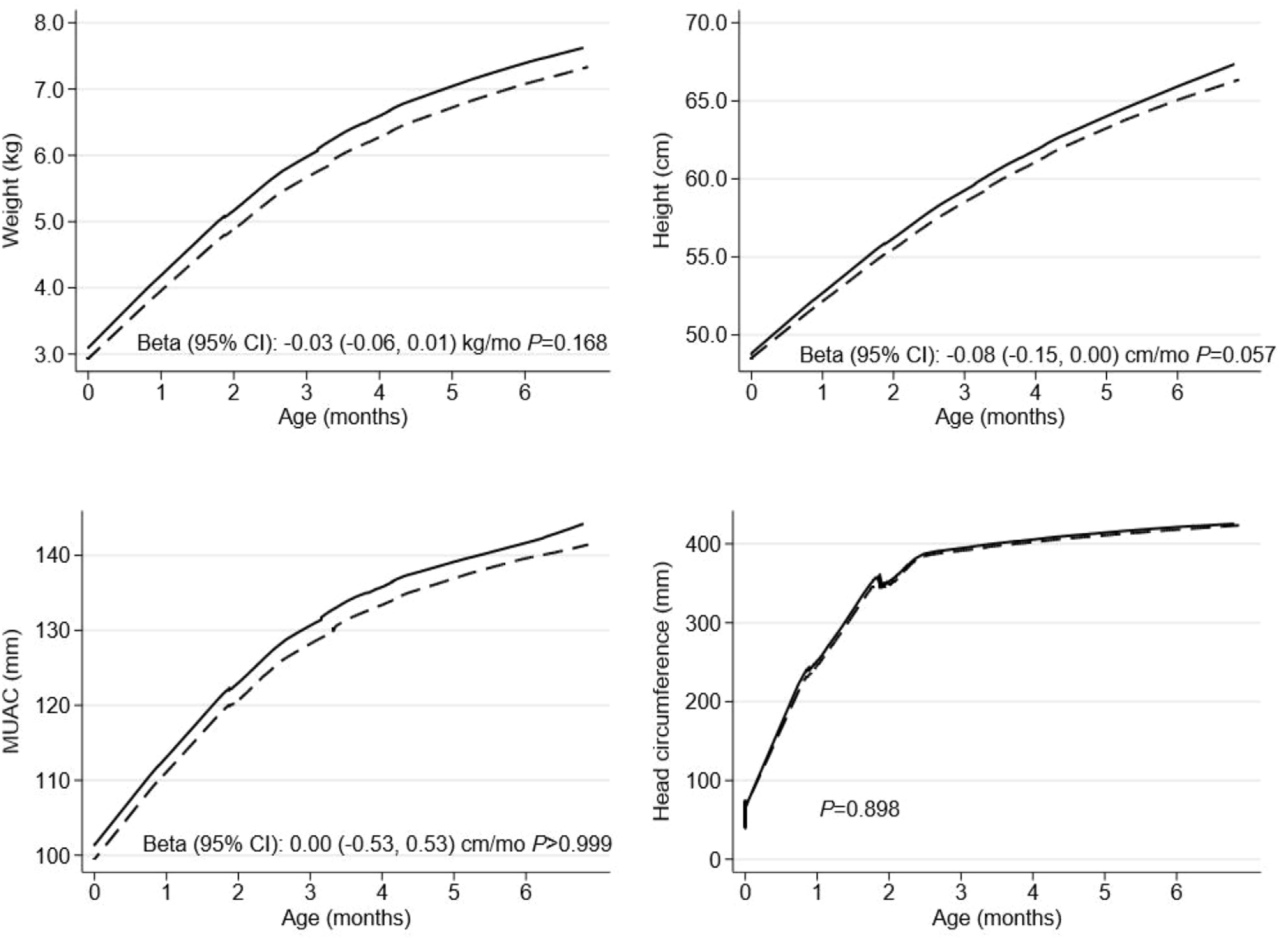
Infant growth trajectories from birth to 6 mo in OTA unexposed (solid lines; *n* = 169) and exposed (dashed lines; *n* = 105) groups. Mixed-effects models with a random intercept for the individual infant and random slope for the child age were fitted to compare OTA-exposed and unexposed groups by growth trajectories during the first 6-mo postpartum. Quadratic models were used for the outcomes height, weight, and MUAC, and a restricted cubic spline model with 4 knots for the outcome head circumference. Fixed effects in the models contained the main effect of time, OTA exposure status, and time-by-exposure interaction, which the later evaluates the difference in monthly growth trajectories between exposed and unexposed groups. Additional covariates in the models included the health center catchment areas, the prenatal and postnatal interventions allocation, maternal age, primiparity, baseline BMI and hemoglobin concentration, household size, wealth index score, access to improved water and sanitation, and food security status. Abbreviations: MUAC, mid-upper arm circumference; OTA, ochratoxin A.

In the unadjusted models, newborn OTA exposure was found to be negatively associated (*P <* 0.05) with birth outcomes, such as birth weight, MUAC, ponderal index, and chest circumference, as well as with LAZ at the age of 6 mo (TABLE 3, TABLE 4). These associations remained significant after adjustment for relevant covariates only for birth weight [adjusted β (95% CI): −0.11 kg (−0.21, 0.00); *P* = 0.042] and ponderal index [adjusted β (95% CI): −0.62 gm/cm^3^ (−1.19, −0.05); *P* = 0.034]. Likewise, newborns who were exposed to OTA had marginally significantly lower length growth trajectories than their counterparts without OTA exposure [adjusted β (95% CI): −0.08 (−0.15, 0.00) cm/mo; *P* = 0.057] (Figure 2).

**TABLE 3:** Newborn ochratoxin A exposure and birth outcomes^1^.

Outcomes	OTA unexposed (*n* = 169)	OTA exposed (*n* = 105)	Unadjusted beta (95% CI)	*P*	Adjusted beta (95% CI)	*P*
Birth weight, (kg)	3.10 ± 0.44	2.95 ± 0.41	−0.15 (−0.26, −0.04)	0.006	−0.11 (−0.21, 0.00)	0.042
Gestational age, (wk)	40.09 ± 1.20	39.84 ± 1.27	−0.23 (−0.54, 0.07)	0.141	−0.18 (−0.49, 0.12)	0.236
Birth length, (cm)	48.73 ± 2.06	48.49 ± 1.94	−0.38 (−0.86, 0.11)	0.125	−0.18 (−0.67, 0.30)	0.453
MUAC, (mm)	101.40 ± 8.24	99.59 ± 8.83	−2.27 (−4.26, −032)	0.023	−1.71 (−3.6, 0.18)	0.077
Head circumference, (cm)	33.43 ± 1.51	33.39 ± 1.38	−0.15 (−0.49, 0.20)	0.403	−0.07 (−0.41, 0.27)	0.705
Ponderal index, (gm/cm^3^)	26.68 ± 2.38	25.80 ± 2.48	−0.66 (−1.24, -0.09)	0.023	−0.62 (−1.19, −0.05)	0.034
Chest circumference, (cm)	32.13 ± 1.62	31.76 ± 1.63	−0.41 (−0.82, −0.01)	0.046	−0.28 (−0.67, 0.11)	0.158
Low birth weight (<2.5 kg)	14 (8.28)	17 (16.19)	6.19 (−1.69, 14.1)	0.123	4.79 (3.28, 12.9)	0.243
Small-for-gestational age	35 (20.71)	34 (32.38)	10.8 (−0.41, 22.0)	0.059	7.80 (−3.33, 18.9)	0.169
Preterm delivery (<37 wk)	3 (1.78)	2 (1.90)	0.09 (−3.26, 3.43)	0.959	−0.17 (−3.85, 3.51)	0.926

Abbreviations: MUAC, mid-upper arm circumference; OTA, ochratoxin A.

1 Values are mean ± SD or frequencies (percentages). Unadjusted and adjusted beta coefficients are estimated using linear regression models for the continuous outcomes and linear probability models with robust variance estimation for the binary outcomes. All models are adjusted for the health center catchment areas and the prenatal and postnatal interventions arms, whereas adjusted models additionally contained maternal age, primiparity, baseline BMI and hemoglobin concentration, and household size, wealth index score, access to improved water and sanitation, and food security status.

**TABLE 4:** Newborn ochratoxin A exposure and infant growth and nutritional status at 6 mo of age^1^.

Outcomes	OTA unexposed(*n* = 169)	OTA exposed(*n* = 105)	Unadjustedbeta (95% CI)	*P*	Adjustedbeta (95% CI)	*P*
Length-for-age z-score	−0.40 ± 1.12	−0.70 ± 1.17	−0.33 (−0.63, –0.03)	0.031	−0.25 (−0.55, 0.05)	0.105
Weight-for-age z-score	−0.34 ± 1.07	−0.62 ± 0.99	−0.22 (−0.50, 0.53)	0.113	−0.17 (−045, 0.10)	0.218
Weight-for-length z-score	−0.03 ± 1.03	−0.20 ± 1.02	−0.08 (−0.34, 0.19)	0.569	−0.07 (−033, 0.20)	0.607
MUAC, (mm)	141.11 ± 11.29	139.08 ± 12.50	−2.42 (−5.17, 0.33)	0.085	−2.18 (−4.97, 0.61)	0.125
Head circumference, (cm)	421.14 ± 15.75	417.84 ± 13.65	−3.37 (−7.18, 0.44)	0.083	−2.90 (−6.76, 0.96)	0.14
Hemoglobin, (g/dL)	10.32 ± 1.31	10.31 ± 1.14	−0.21 (−0.52, 0.10)	0.187	−0.20 (−0.52, 0.11)	0.204
Stunting	13 (8.23)	10 (10.20)	0.68 (−6.81, 8.16)	0.859	−0.11 (−8.23, 8.00)	0.978
Underweight	7 (4.43)	7 (7.22)	2.00 (−4.53, 8.54)	0.546	2.19 (−4.82, 9.20)	0.539
Wasting	3 (1.90)	3 (3.09)	1.28 (−2.74, 5.30)	0.531	1.53 (−2.57, 5.63)	0.464
Anemia	108 (69.23)	67 (70.53)	6.74 (−4.93, 18.4)	0.257	6.79 (−4.87, 18.4)	0.252

Abbreviations: MUAC, mid-upper arm circumference; OTA, ochratoxin A.

1 Values are mean ± SD or frequencies (percentages). Unadjusted and adjusted betas are estimated using linear regression models for the continuous outcomes and linear probability models with robust variance estimation for the binary outcomes. All models are adjusted for the health center catchment areas and the prenatal and postnatal interventions arms, whereas adjusted models additionally contained maternal age, primiparity, baseline BMI and hemoglobin concentration, and household size, wealth index score, access to improved water and sanitation, and food security status.

There was also a significant interaction between newborn OTA exposure status and the maternal prenatal BEP intervention on the outcome of child anemia status at 6 mo of age (*P-*interaction = 0.074) (Supplemental Table 1). OTA exposure was significantly associated with higher anemia prevalence among newborns of mothers who did not receive prenatal BEP supplementation [adjusted β (95% CI): 18.7% (2.00, 35.3); *P* = 0.029], whereas no significant association was detected among newborns whose mothers received the prenatal BEP supplementation [adjusted β (95% CI): 0.08% (−19.1, 19.3); *P* = 0.993]. In the present study, there was a 63.0% agreement between mother–newborn OTA exposure status, and the kappa value was classified as fair (Kappa = 0.27) (Supplemental Table 2).

## Discussion

There is a growing concern about the potential adverse health and developmental consequences of fetal mycotoxins exposure. The present study found a high prevalence of OTA exposure (38.32%) among newborns at birth. Exposures to mycotoxins, such as AFG1, AFB1, AFM1, CIT, DON, zearalanone, and ZEN were detected in relatively fewer subjects, whereas other mycotoxins were not detected. Moreover, we found that OTA-exposed newborns had significantly lower birth weight and ponderal index than their non-exposed counterparts. OTA-exposed newborns also had marginally significantly lower length growth trajectories. Finally, an exploratory analysis indicated that maternal prenatal BEP supplementation may offset the effect of OTA exposure on increased anemia prevalence at 6 mo of age.

OTA is a toxin produced by *Aspergillus ochraceus, A*. *niger, A*. *carbonarius, A*. *sulphureus, Penicillium viridicatum, P*. *cyclopium,* and other species. This toxin widely contaminates staple foods. Given the dietary habits of the study population, where nearly all participants regularly consume tô, a staple maize dough, the Burkinabé population is often exposed to OTA [[42], [43], [44]]. Similarly, other research in Burkina Faso has found OTA contamination in a range of products, including commercial infant formulas, maize, groundnuts, sorghum, millet, rice, wheat, soy, dried fruits, processed foods, and animal feeds [45,46].

The OTA occurrence detected in the present study is similar to findings from other studies using adult samples, where OTA was found in 28% of cases with concentrations ranging from 0.31 μg/L to 11.67 μg/L [19,20]. In contrast, other studies reported higher OTA occurrences, ranging from 96% to 100%, with concentrations between 0.1 μg/L and 6.63 μg/L [18,47]. However, exposure to mycotoxins other than OTA was found to be low in our study population as compared with what has been reported previously in LMICs [21,[48], [49], [50]]. The variations in the physicochemical properties of mycotoxins can lead to differences in their toxicokinetic profiles, which results in different excretion amounts and times. Therefore, it is conceivable that the UPLC-MS/MS used system may not detect the lowest concentrations of mycotoxin metabolites [51]. Additionally, the knowledge of the formation process of these metabolites and the understanding of their structure and molecular mass can solve the analytical and technological challenges associated with these metabolites. Further research is needed to understand the stability of mycotoxins during food processing, their behavior in the digestive system, and their toxicodynamic and toxicokinetic properties. Consequently, due to these factors, exposure to certain mycotoxins may be underreported because of their short half-lives.

In the present study, results indicated a reduction of 0.11 kg in birth weight in newborns exposed to OTA compared with unexposed newborns, though previous findings that OTA exposure is associated with poor birth outcomes and infant growth have been reported inconsistently. For example, Jonsyn et al. (1995) [14] reported that the presence of OTA combined with AFs and their metabolites in cord blood samples was likely to reduce birth weight. Similarly, a study in Bangladesh found that maternal dietary intake of OTA was associated with higher odds of having an LBW infant, with the risk increasing in a dose-dependent manner [15]. Additionally, research in Ethiopia identified a link between chronic maternal AF exposure and reduced fetal growth trajectories, as measured by fetal biometry through ultrasound estimates, although this study did not find a correlation between AF exposure and birth anthropometry [17]. Given the wide variation in detected mycotoxin concentrations and possible confounding factors adjusted for in these studies, it is not surprising that some found associations and others did not.

In contrast to the present study, in our previous analysis of OTA exposure during the third trimester of pregnancy in the same cohort, we did not find associations between maternal exposure and growth at birth and at 6 mo of age [32]. Besides this, we only found a fair level of agreement between maternal OTA exposure during the third trimester of pregnancy (50.8%) and neonatal exposure (38.3%) status (Kappa = 0.27). There was also no constant pattern in the type or quantity of OTA detected in maternal and newborn samples in other studies. A study in Sierra Leone detected 12.5% OTA exposure in maternal serum samples compared with 25.5% in cord blood samples [8]; whereas a study in Bolivia detected OTA in 87% in the cord plasma samples compared with 12.5% in the maternal plasma samples [52]. A possible explanation for the higher detection of OTA in the previous analysis of prenatal maternal OTA exposure [32] is that it was conducted at 30–34 wk of gestation. Previous literature suggests that OTA transfer from the mother to the fetus occurs at a higher rate in the earlier stages of pregnancy than in the later stages [53]. Furthermore, OTA distribution in the human body could also be affected by the development of the placenta and physiological differences throughout pregnancy [54]. Lastly, there could be seasonal variations in mycotoxin exposure status depending on food availability, and storage conditions since the maternal samples were collected between July and March [32] whereas the newborn samples were collected between May and October.

In the earlier study by de Kok et al. (2022) [55] maternal BEP supplementation during pregnancy and lactation was beneficial in reducing the prevalence of LBW and improving GA, birth weight, birth length, and chest circumference. However, iron and folic acid supplementation in the form of BEP or IFA tablet formulations did not improve anemia prevalence during pregnancy [56]. Similarly, there was a high prevalence of infant anemia at 6 mo of age in both intervention and control groups [57] suggesting the limited effect of maternal iron and folic acid supplementations in the form of BEP and IFA tablets formulations. On the other hand, the exploratory analysis in this study indicated a beneficial role of BEP in mitigating the negative effect of OTA exposure on increased infant anemia at the age of 6 mo. To the best of our knowledge, there is no other study addressing the role of nutritional supplementation on the effects of mycotoxins exposure.

Considering the risks posed by OTA and other mycotoxins in LMICs, Matumba et al. (2021) [58] proposed a framework for the prevention and control of mycotoxins in grains. The guideline has 5 pointers including the following: *1*) Sustaining the plant’s strength and health; *2*) Reducing toxigenic fungal population in growing plants and storage; *3*) Rapidly reducing the moisture content of grains and avoiding rehydration; *4*) Safeguarding the outer structure of seeds/grains; and *5*) Cleaning and removing mycotoxin high-risk components. The guideline also provides recommendations on how grains should be handled from production, harvesting, and storage practices to processing considering the factors that promote or prevent fungal contamination and subsequent production of mycotoxins in grains [58].

Key strengths of the present study include determination of GA using ultrasonography and the assessment of birth outcomes within 12 h of birth, allowing the timely and robust assessment of study outcomes [59]. This is also the first application of VAMS for mycotoxin analysis in the whole blood of newborns in an LMIC setting, utilizing biomarkers to determine mycotoxin exposure offers a more accurate assessment compared with evaluating foodstuffs, as done in some other studies. Considering the other benefits of VAMS and the robust method developed, VAMS sampling can be considered as an alternative technique to perform a quantitative screening of mycotoxin exposure [40]. The findings also provide support for future studies, using larger cohorts, with sampling conducted through VAMS. In addition, considering the toxicokinetic profiles of the detected mycotoxins, this microsampling technique will further highlight the effect of exposure to mycotoxins on human health, enabling further associations to be made with adverse health outcomes. Lastly, as a limitation, mycotoxin exposure data from only a single time point postnatally was considered. Future studies, using repeated mycotoxins measurements, will provide an insight into the effects of mycotoxins and their physicochemical properties in relation to the timing of exposure, and also whether the contamination in the maternal food supply changes. Moreover, further studies assessing mycotoxin exposure during the complementary feeding period in infants and young children will also provide a full picture of the burden of the problem and its effects during the critical window period in this and similar populations. It should be also noted that our analysis was mainly exploratory evaluating associations between OTA exposure and multiple newborn and infant outcomes, therefore we cannot rule out the possibility of multiple testing effects in our positive results.

In conclusion, this study reports a high occurrence of newborn OTA exposure and an associated risk of lower birth weight, ponderal index, and length growth trajectories in rural Burkina Faso. The findings emphasize the importance of nutrition-sensitive strategies to mitigate dietary OTA in the food supply, as well as adopting food safety measures in LMICs during the fetal period of development.

## Author contributions

The authors’ responsibilities were as follows – YB-M, AA, TD-C, SDS, CL, MDB: conceptualized the study; YB-M, TD-C, SDS, CL, MDB: designed the study; YB-M, AA, TD-C: designed the software used in the study; YB-M, GDP, JE-H, SDS, MDB: validated the study; YB-M, AA: formally analyzed the study; YB-M, AA, GDP, TD-C, JE-H, LO, SDS, MDB: investigated the study; YB-M, TD-C, LO, LCT, SDS, CL, MDB: organized the resources; YB-M, AA, TD-C, LO: curated the data; YB-M, AA: wrote the original draft; YB-M, AA, GDP, TD-C, JE-H, LO, LCT, SDS, CL, MDB: reviewed and edited the draft; TD-C, SDS, CL, MDB: supervised the study; YB-M, TD-C, LO, LCT, SDS, CL and MDB: administrated the study; TD-C, CL, MDB: acquired the funding; and all authors: read and approved the final manuscript.

## Funding

The MISAME-III main trial work was supported by the Bill & Melinda Gates Foundation (OPP1175213). The mycotoxins sampling and analysis were supported by Fonds Wetenschappelijk Onderzoek (Project No. G085921N). MDB was supported by the European Research Council under the European Union’s Horizon 2020 research and innovation program (Grant Agreement No. 946192, HUMYCO). The funders had no role in the design and conduct of the study; collection, management, analysis, and interpretation of the data; and preparation, review, or approval of the manuscript.

## Data availability

Data will be made available through a data-sharing agreement. Please contact carl.lachat@ugent.be and marthe.deboevre@ugent.be for any queries. Supporting study documents, including the study protocol and questionnaires, are publicly available on the study’s website: https://misame3.ugent.be (accessed on 07 December 2023).

### Consent for publication

All the authors also agreed on the publication of this article.

### Consent to participate

Informed consent was obtained from all subjects involved in the study.

## Ethical considerations

The study protocol was approved by the Ethical Committee of Ghent University Hospital in Belgium (B670201734334) and the Ethical Committee of Institut de Recherche en Sciences de la Santé in Burkina Faso (50-2020/CEIRES). Written informed consent was obtained from all subjects involved in the study.

## Conflicts of interest

The authors report no conflicts of interest.
