# Supplementary material for: Ochratoxin A Status at Birth Is Associated with Reduced Birth Weight and Ponderal Index in Rural Burkina Faso
Source: J Nutr. 2024 Oct 10;155(1):260–9. doi: 10.1016/j.tjnut.2024.10.015 (PMC11795690; doi:10.1016/j.tjnut.2024.10.015)
Supplement: multimedia component 1 [file mmc1.docx]

**Supplemental Table 1: Infant anemia status at 6 months of age by OTA exposure status for the whole study sample and by maternal prenatal supplementation groups^1^**

| **Outcomes** | **OTA unexposed** | **OTA exposed** | **Unadj beta (95% CI)** | ***p*** | **Adj beta (95% CI)** | ***p*** |
| --- | --- | --- | --- | --- | --- | --- |
| **Overall sample (n = 251)** |  |  |  | 0.070^2^ |  | 0.074^2^ |
| Anemia | 108 (69.2) | 67 (70.5) | 6.74 (-4.93, 18.4) | 0.257 | 6.79 (-4.87, 18.4) | 0.252 |
| **IFA group (n = 135)** |  |  |  |  |  |  |
| Anemia | 57 (69.5) | 42 (79.3) | 16.3 (0.95, 31.6) | 0.038 | 18.7 (2.00, 35.3) | 0.029 |
| **BEP + IFA group (n = 116)** |  |  |  |  |  |  |
| Anemia | 51 (68.9) | 25 (59.5) | -4.01 (-21.8, 13.8) | 0.657 | 0.08 (-19.1, 19.3) | 0.993 |

^1^Linear probability models with robust variance estimation were used to determine the adjusted and unadjusted differences in anemia status in percentage points and their associated *p*-values. In the model analyzing the overall sample, the interaction between OTA exposure and the maternal prenatal nutritional supplementation was evaluated by introducing interaction terms with *p*-values^2^ <0.10 considered as significant interactions. All models are adjusted for the health center catchment areas and the prenatal and postnatal interventions arms, while adjusted models additionally contained maternal age, primiparity, baseline BMI and hemoglobin concentration, and household size, wealth index score, access to improved water and sanitation, and food security status. BEP, balanced energy-protein; IFA, iron-folic acid; OTA, ochratoxin A
