# Supplementary material for: Ochratoxin A Status at Birth Is Associated with Reduced Birth Weight and Ponderal Index in Rural Burkina Faso
Source: J Nutr. 2024 Oct 10;155(1):260–9. doi: 10.1016/j.tjnut.2024.10.015 (PMC11795690; doi:10.1016/j.tjnut.2024.10.015)
Supplement: multimedia component 2 [file mmc2.docx]

**Supplemental Table 2: Agreement between maternal OTA exposure during trimester 3 and newborn OTA exposure at birth**

| **Maternal OTA status** | **Newborn OTA status** | | **Total** |
| --- | --- | --- | --- |
|  | **Exposed: n (%)** | **Unexposed: n (%)** |  |
| **Exposed** | 98 (75.38) | 32 (24.62) | 130 |
| **Unexposed** | 68 (48.57) | 72 (51.43) | 140 |
| **Total** | 166 (61.48) | 104 (38.52) | 270 |

OTA, ochratoxin A
